# Surface proteins of *Propionibacterium freudenreichii* MJ2 inhibit RANKL-induced osteoclast differentiation by lipocalin-2 upregulation and lipocalin-2-mediated NFATc1 inhibition

**DOI:** 10.1038/s41598-023-42944-y

**Published:** 2023-09-20

**Authors:** Jiah Yeom, Seongho Ma, Dong Joon Yim, Young-Hee Lim

**Affiliations:** 1 Department of Integrated Biomedical and Life Sciences, Graduate School, Korea University, Seoul, 02841 Republic of Korea; 2 School of Biosystems and Biomedical Sciences, Korea University, Seoul, 02841 Republic of Korea; 3 Department of Laboratory Medicine, Korea University Guro Hospital, Seoul, 08308 Republic of Korea

**Keywords:** Microbiology, Rheumatology

## Abstract

Osteoclasts degrade bone and osteoclast differentiation has been implicated in bone destruction in rheumatoid arthritis. The dairy bacterium *Propionibacterium freudenreichii* MJ2 (MJ2) isolated from raw milk inhibits osteoclast differentiation and ameliorates collagen-induced arthritis. This study aimed to investigate the inhibitory effect of the surface proteins of MJ2 on receptor activator of nuclear factor-κB ligand (RANKL)-induced osteoclast differentiation and explain the underlying mechanism. The murine macrophage cell line RAW 264.7 was used to study the inhibition of osteoclast differentiation. The surface proteins significantly inhibited RANKL-induced osteoclast differentiation in a protein concentration-dependent manner by inhibiting the expression of genes and proteins related to osteoclast differentiation. RNA microarray analysis showed that the surface proteins significantly upregulated lipocalin-2 (lcn2) expression. In addition, they downregulated c-fos and NFATc1 and inhibited the expression of NFATc1-downstream genes *Atp6v0d2*, *Calcr,* and *Ctsk*. siRNA silencing of lcn2 decreased the extent of surface protein inhibition on osteoclast differentiation, suggesting that lcn2 plays an important role in the inhibition of RANKL-induced osteoclast differentiation. In conclusion, surface proteins of MJ2 show inhibitory effects on RANKL-induced osteoclast differentiation by upregulating lcn2 expression, in turn downregulating NFATc1, leading to the inhibition of NFATc1-downstream osteoclastogenesis-related gene expression.

## Introduction

Rheumatoid arthritis (RA) is an inflammatory disease that could occur in all age groups. RA is known as an autoimmune disease, however, the exact cause of RA is unknown^1,2^. RA activates synovial cells in the joints and induces inflammation, cartilage destruction, and further bone damage. Recently, the involvement of osteoclasts in bone erosion in RA was reported^3^. In patients with RA, osteoclasts located in the synovium and bone interface erode the joint bone at an early stage of the disease and gradually worsen bone loss^3^.

Osteoclasts, the major bone-destroying cells, are multinucleated cells that differentiate from the monocyte-macrophage lineage. In this process, receptor activator of nuclear factor-κB (RANK) ligand (RANKL) attaches osteoclast precursor cells to the bone matrix and then activates the differentiation of immature osteoclasts into a mature state^4^. Mature steoclasts create an acidic bone microenvironment, and secrete substances that mediate bone destruction, including tartrate-resistant acid phosphatase (TRAP), cathepsin K (Ctsk), and calcitonin receptor (Calcr). In addition, osteoclasts secret pro-inflammatory cytokines and chemokines, which proceeds the inflow of more osteoclast precursors; eventually, inflammation and bone destruction worsen with a consequent RA process. Therefore, an intensive study focusing on osteoclasts is needed to understand RA development in detail.

Osteoclasts in the RA microenvironment are more easily differentiated from macrophages by increasing the RANKL to osteoprotegerin (OPG) ratio^5^. The OPG/RANKL/RANK system between osteoblasts and osteoclasts becomes a new target to improve bone diseases, such as RA and osteoporosis^5^. RANKL, a major factor in joint destruction, binds to RANK expressed on osteoclast precursors and then induces osteoclast differentiation. Thus, chemical and biological agents inhibiting RANKL expression have been in the spotlight as therapeutic agents for preventing joint destruction in patients with RA^4^.

To date, no drugs can completely cure RA and most drugs are only focused on the attenuation of inflammation. Alternatively, steroids, nonsteroidal anti-inflammatory drugs, and disease-modifying anti-rheumatic drugs, are used to improve RA symptoms by delaying the progression of RA or relieving pain and inflammation. Consumption of them are known to have side effects, such as liver injury and development of tuberculosis^6,7^. A few agents target the inhibition of osteoclast differentiation, specifically inhibiting the RANKL-RANK interaction. Bisphosphonates and zoledronic acid have been used to inhibit osteoclast differentiation^8,9^. Denosumab, an anti-RANKL antibody, blocks RANKL-RANK binding, thereby inhibiting osteoclast differentiation and preventing RA-associated bone erosion, although it does not prevent inflammation^10^. Therefore, safer and more effective agents for preventing osteoclast differentiation and bone loss should be developed to treat RA.

Recently, probiotics receive a lot of attention as a treatment for autoimmune diseases such as RA because of their beneficial effects on the immune system and gut-bone axis^11,12^. A dairy *Propionibacterium freudenreichii* is a beneficial bacterium possessing probiotic properties, including anti-inflammatory and bifidogenic activities^13,14^. In our previous study, we observed that heat-killed *P. freudenreichii* MJ2 (MJ2) isolated from raw milk promotes osteoblast differentiation and alleviates osteoporosis by increasing the OPG to RANKL ratio^15^. In addition, MJ2 inhibited RANKL-induced osteoclast differentiation^16^. Based on our previous study, we separated surface, secreted, and cytoplasmic proteins from *P. freudenreichii* MJ2 and found that surface proteins showed inhibitory effects on osteoclastogenesis. Therefore, the aim of this study was to investigate the effect of surface proteins of MJ2 on RANKL-induced osteoclast differentiation and to elucidate the inhibitory mechanism of osteoclast differentiation.

## Results

### Cytotoxicity of surface proteins isolaed from *P. freudenreichii* MJ2 in RAW 264.7 cells

The number of dead bacteria stained with propidium iodide (PI) did not significantly increase in MJ2 treated with Gu-HCl compared with the number of live MJ2 without Gu-HCl treatment (Supplementary Fig. 1). The results indicate that chaotropic agent (guanidine-HCl (Gu-HCl)) used for extracting surface proteins from MJ2 did not significantly damage bacterial cells, which means that the extracted surface proteins contained few proteins derived from the cytoplasm by cell lysis. Therefore, we used surface proteins extracted by Gu-HCl treatment in this study.

The viability of the cells treated with surface proteins up to 10 μg/mL did not show any cytotoxicity, moreover, the viability of the cells treated with 2.5 and 5 μg/mL surface proteins showed a significant increase (Supplementary Fig. 2). Therefore, we used surface proteins at concentrations of 2.5, 5, and 10 μg/mL.

### Surface proteins inhibit TRAP activity in RAW 264.7 cells

**Figure 1:**
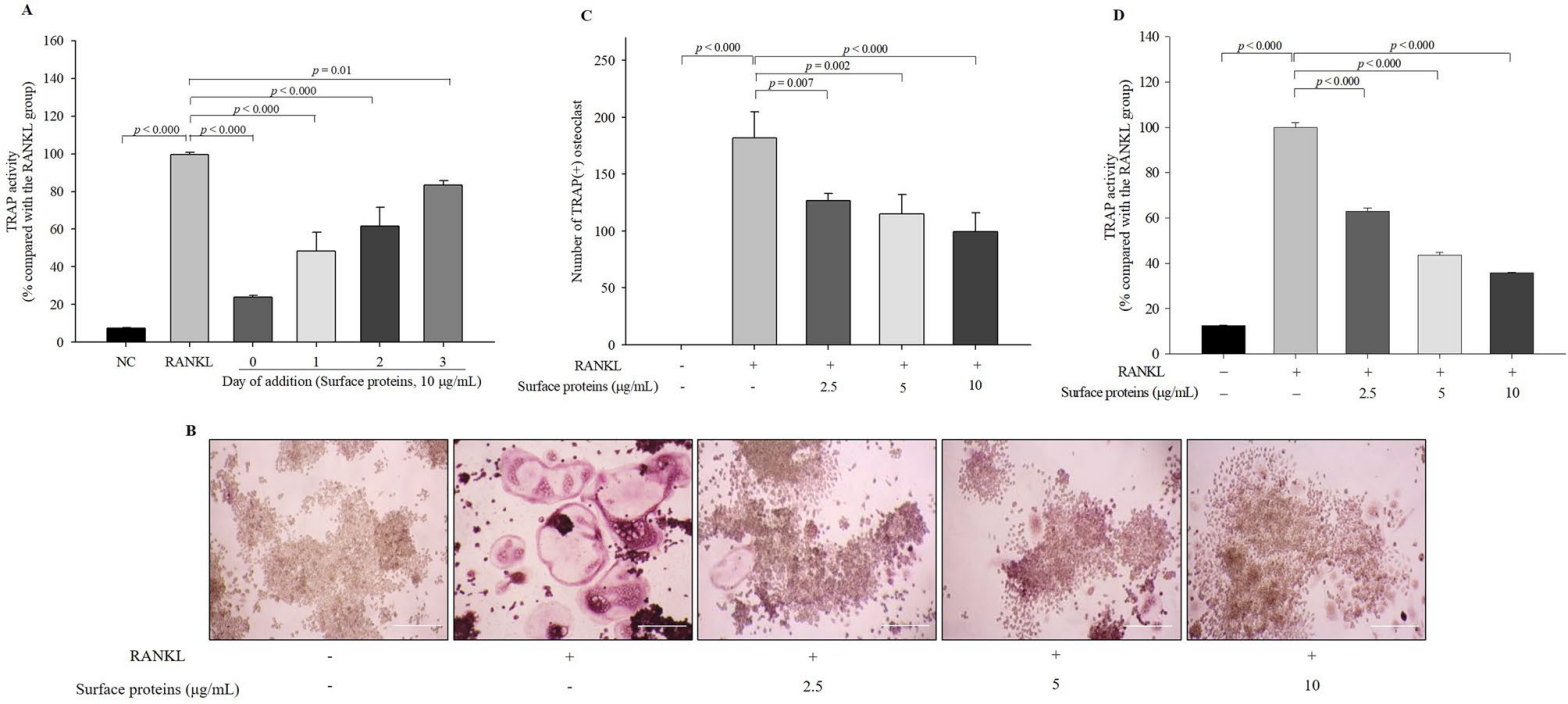
Effect of surface proteins on osteoclastogenesis. Surface proteins were added immediately (0 day) or after 1, 2, or 3 days to the RANKL-treated cells and TRAP(+) osteoclast number and activity were measured on Day 4 after treatment of RANKL (**A**). RANKL-induced osteoclast formation was measured by TRAP staining after 4 days of treatment of surface proteins (**B**) (100 × , scale bar = 100 μm) and the number of TRAP(+) osteoclasts (**C**) and TRAP activity (**D**) were quantified. The values indicate the mean ± SD of three independent experiments performed in triplicates.

Differentiation of osteoclast precursor cells into mature osteoclasts takes time; accordingly, RANKL-induced osteoclast differentiation from RAW 264.7 cell is clearly observed after 3 days^17^. Thus, to determine whether surface proteins affect the early or late stages of osteoclastogenesis, surface proteins (10 μg/mL) were added immediately (0 day) or after 1, 2, or 3 days to the RANKL-treated cells, and TRAP activity was measured after 4 days of RANKL-induction. All the results obtained for each addition of surface proteins showed a significant effect on the inhibition of osteoclast differentiation, although the degree of inhibitory effect gradually decreased in a time-dependent manner (Fig. 1A). These results suggest that surface proteins inhibit osteoclast differentiation during the early stages of osteoclastogenesis.

After 4 days of treatment, TRAP(+) osteoclast number and activity in the surface protein-treated cells significantly decreased in a protein concentration-dependent manner compared with the cells treated with RANKL alone (Fig. 1B–D). These results suggest that surface proteins inhibited osteoclastogenesis and TRAP activity, which means that surface proteins contain the active component(s) of MJ2 that inhibits osteoclast differentiation.

### Surface proteins decrease the expression levels of osteoclast-related genes and proteins

**Figure 2:**
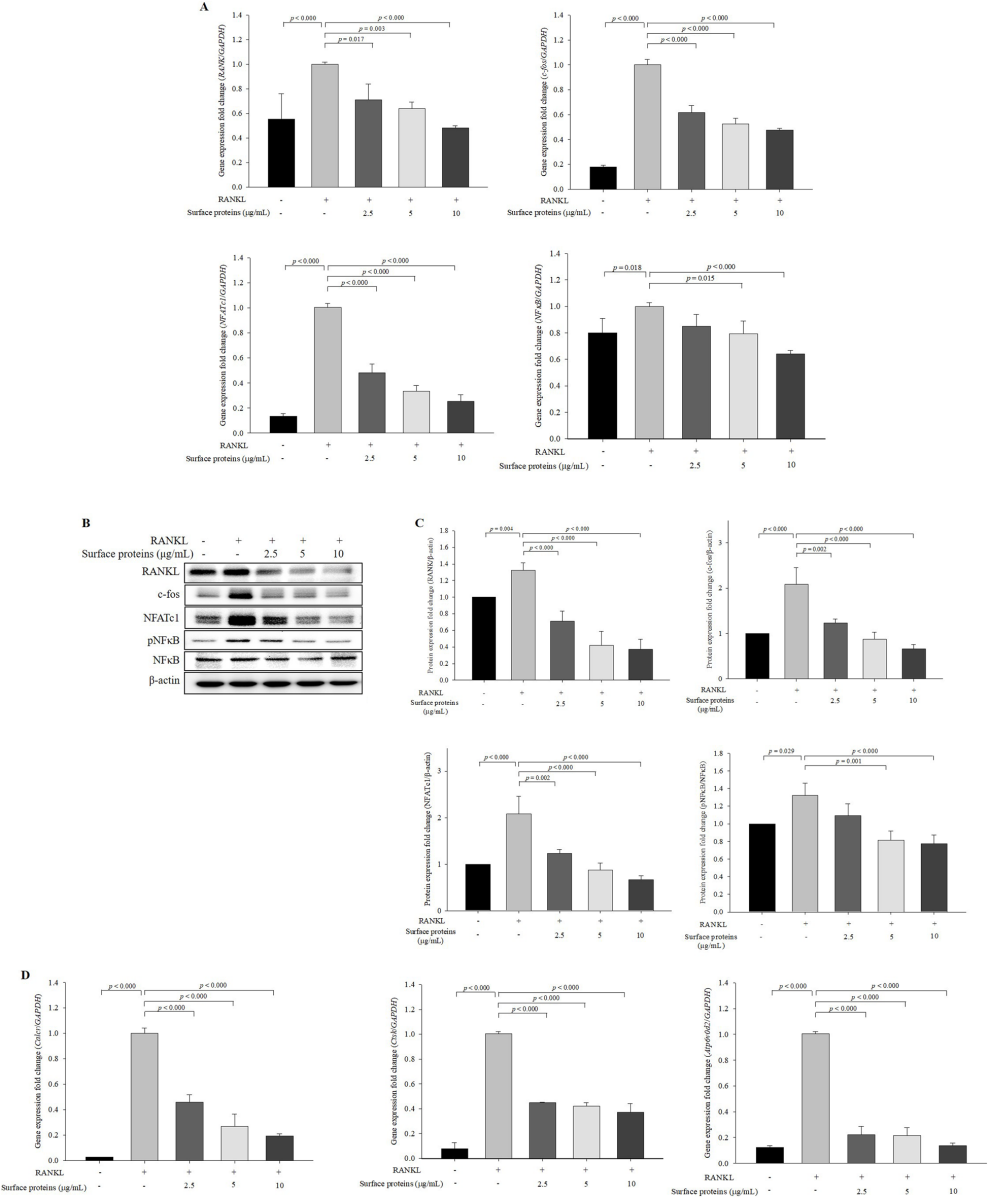
Expression levels of osteoclast differentiation-related genes and proteins in the cells treated with surface proteins extracted from *P. freudenreichii* MJ2. The expression levels of RANKL-induced osteoclastogenic genes proteins were measured by qPCR and western blot, respectively. RANKL-induced osteoclastogenic genes (**A**), proteins (**B**) and quantified (**C**), and NFATc1-downstream genes (**D**). The values indicate the mean ± SD of three independent experiments performed in triplicates. The full-length blots are shown in Supplementary Fig. 4.

The expression levels of RANKL-induced osteoclastogenic genes, including *RANK*, *c-fos*, *NFATc1,* and *NFκB*, significantly decreased in cells treated with surface proteins in a protein concentration-dependent manner compared with those in the cells treated with RANKL alone (Fig. 2A). At the protein level, the expression levels of NFATc1 (a master transcription regulator of osteoclast differentiation), c-fos (a key regulator of osteoclast-macrophage lineage determination), RANK, and pNFκB/NFκB significantly decreased in the cells treated with the surface proteins in a protein concentration-dependent manner compared with the those in the cells treated with RANKL alone (Fig. 2B,C). The expression levels of NFATc1-downstream genes, such as *Calcr*, *Ctsk,* and *Atp6v0d2* (ATPase, H + transporting, lysosomal V0 subunit D2), which is a subunit associated with proton transport in the plasma membranes of osteoclasts, were significantly decreased in the cells treated with surface proteins compared with those in the cells treated with RANKL alone (Fig. 2D). These results suggest that surface proteins inhibited RANKL-induced osteoclast differentiation.

### Gene expression analysis of surface protein-treated cells

**Figure 3:**
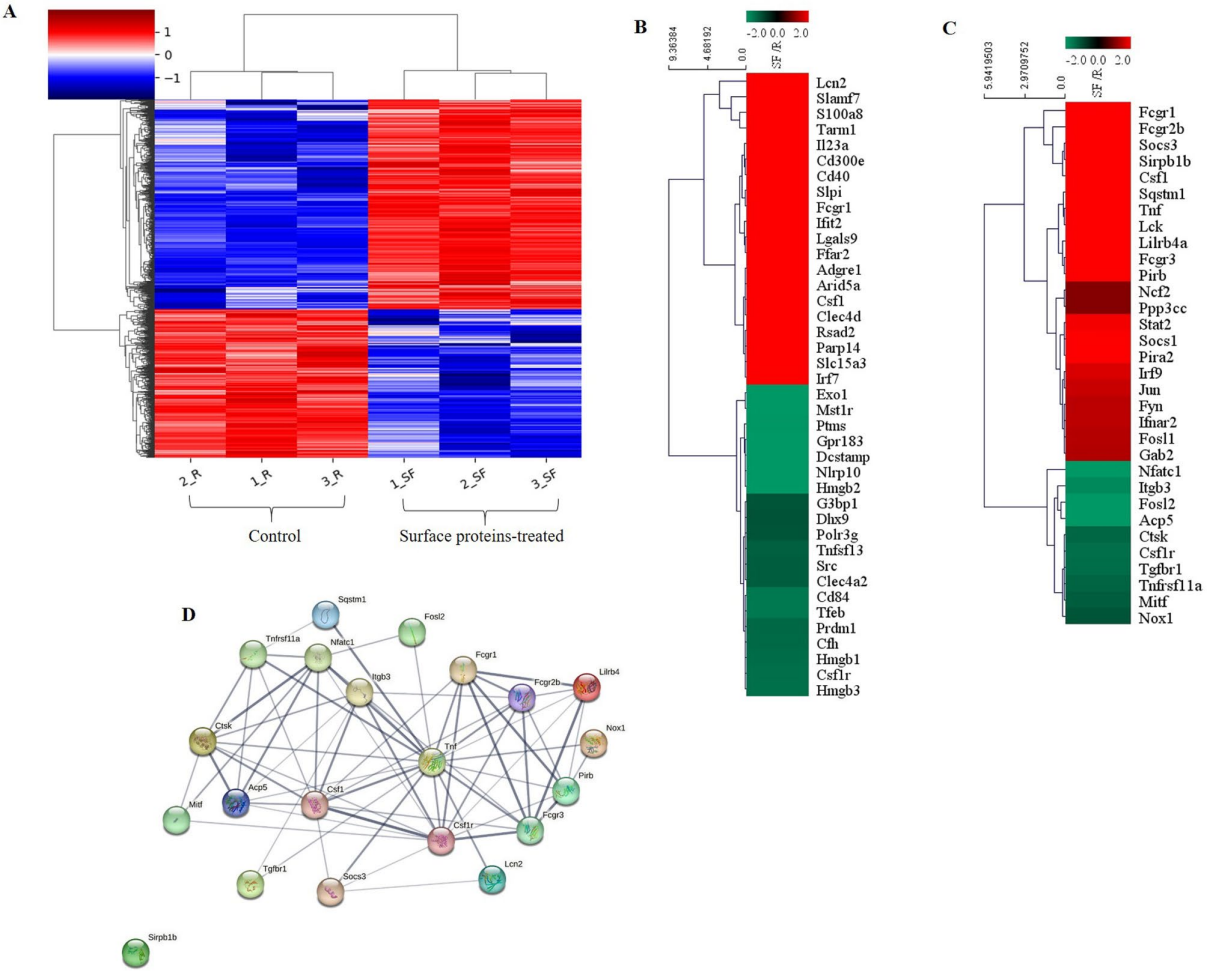
Results of the RNA sequencing of cells treated with surface proteins of *P. freudenreichii* MJ2. The differentially expressed genes (DEGs) between the negative control and surface proteins-treated cells were analyzed with excel-based differentially expressed gene analysis (ExDEGA). (**A**) Heatmap of hierarchically clustered samples/genes (red, significantly upregulated; blue, significantly downregulated). (**B**) Heatmap based on the data of the 40 genes with 20 highest and lowest expression levels (fold change ≥ 2 or ≤ 0.5, *p* < 0.05) in immune system process function (red, significantly upregulated; green, significantly downregulated). (**C**) Heatmap based on the data of osteoclast differentiation-related genes (32 genes) with expression level to fold change ≥ 2 or ≤ 0.5 (*p* < 0.05) (red, significantly upregulated; green, significantly downregulated). (**D**) Interaction network of 21 genes (lcn2 along with 20 genes with 10 highest and lowest expression levels in C, Table 1) identified by Search Tool for the Retrieval of Interacting Genes/Proteins (STRING) analysis with a confidence cutoff of 0.40 using the STRING database. In the resulting gene association network, genes are presented as nodes connected by lines whose thickness represents the confidence level.

RNA sequencing of the negative control sample treated with RANKL alone and that of the cells treated with surface proteins and RANKL were performed to explore the inhibitory mechanism of surface proteins in osteoclast differentiation, and a total of 23,282 genes were identified. Among the total genes identified, 888 genes showed a significant increase (more than twofold) or decrease (less than 0.5-fold) (*p* < 0.05) in the expression level compared with those in the negative control. Using these genes, hierarchical clustering was performed among the groups of samples/genes and represented as heatmap (Fig. 3A). The dendrograms on the top and left sides show similar expression patterns among samples and genes, respectively. The results show that genes with similar expression patterns were relatively consistent within the control or surface protein-treated samples. Using these 888 genes, Kyoto Encyclopedia of Genes and Genomes (KEGG) enrichment analysis was performed, and the Database for Annotation, Visualization, and Integrated Discovery (DAVID) showed that the genes involved in the immune system process included the most upregulated genes. The heatmap based on the data of the 40 genes with 20 highest and lowest expression levels (fold change ≥ 2 or ≤ 0.5, *p* < 0.05) in the immune system process function showed that *lcn2* (lipocalin 2) was the most highly expressed gene (2426-fold) (Fig. 3B). In addition, 128 osteoclast differentiation-related genes were found and among them, of which 32 genes, including *Nfatc1*, *Acp5*, *Tnfrsf11a*, and *Ctsk* showed a fold change of ≥ 2 or ≤ 0.5 (*p* < 0.05) (Fig. 3C). To investigate the relationship between osteoclast differentiation-related genes and lcn2, interactions between lcn2 and 20 osteoclast differentiation-related genes (10 genes each with highest and lowest expression levels) were predicted using STRING analysis with the minimum required interaction score of 0.400 at medium confidence (Table 1). Lcn2 directly interacts with tumor necrosis factor (Tnf) which directly interacts with *Tnfrsf11a* (RANK gene) and *Nfatc1* followed by Nfatc1-downstrem factors including *Cst1, Ctsk*, *Mitf*, and *Acp5* (TRAP gene) (Fig. 3D). By referring to previously reported result^18^, we hypothesized that lcn2 might be involved in the inhibitory mechanism of surface proteins on osteoclast differentiation.

**Table 1:** Genes used for STRING analysis.

Gene symbol	Gene description	Fold change	*p* value
*Lcn2*	lipocalin-2	2425.546	0.036
*Fcgr1*	Fc receptor, IgG, high affinity I	61.600	≤ 0.000
*Fcgr2b*	Fc receptor, IgG, low affinity IIb	27.985	≤ 0.000
*Socs3*	suppressor of cytokine signaling 3	15.981	0.001
*Sirpb1b*	signal-regulatory protein beta 1B	15.498	0.010
*Csf1*	colony stimulating factor 1 (macrophage)	13.331	0.042
*Sqstm1*	sequestosome 1	6.045	0.002
*Tnf*	tumor necrosis factor	5.438	≤ 0.000
*Lck*	lymphocyte protein tyrosine kinase	5.081	0.017
*Lilrb4a*	leukocyte immunoglobulin-like receptor, subfamily B, member 4A	7.638	0.003
*Fcgr3*	Fc receptor, IgG, low affinity III	8.545	0.021
*Nfatc1*	Nuclear factor of activated T cells	0.125	≤ 0.000
*Itgb3*	integrin beta 3	0.288	0.023
*Fosl2*	fos-like antigen 2	0.220	≤ 0.000
*Acp5*	acid phosphatase 5, tartrate resistant	0.170	0.003
*Ctsk*	cathepsin K	0.390	0.023
*Csf1r*	colony stimulating factor 1 receptor	0.365	≤ 0.000
*Tgfbr1*	transforming growth factor, beta receptor I	0.368	0.007
*Tnfrsf11a*	tumor necrosis factor receptor superfamily, member 11a, NFKB activator	0.407	≤ 0.000
*Mitf*	microphthalmia-associated transcription factor	0.425	≤ 0.000
*Nox1*	NADPH oxidase 1	0.462	0.002

### Inhibition of lcn2 decreases the inhibitory effect of surface proteins on TRAP activity and actin ring formation

**Figure 4:**
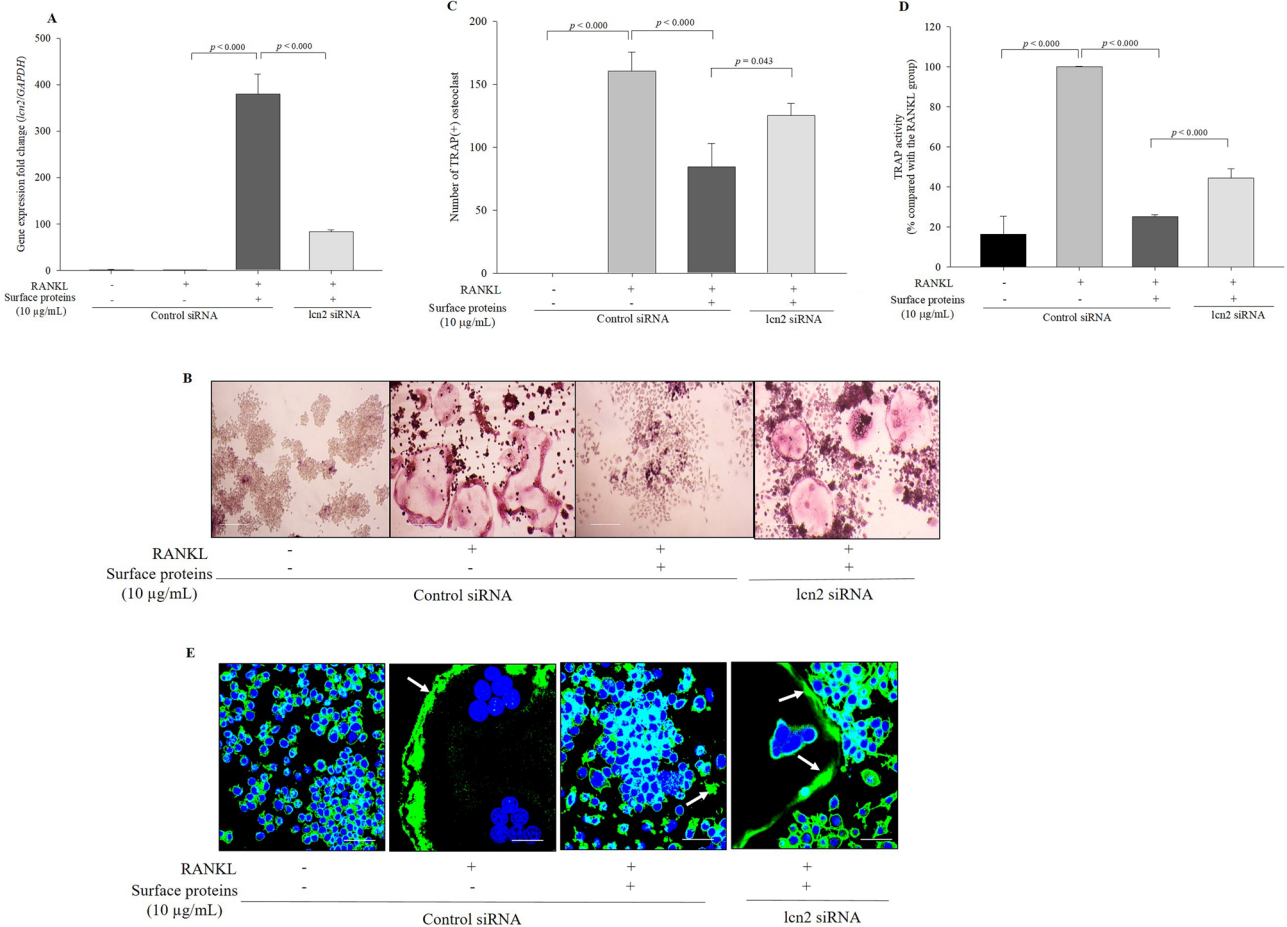
Inhibition of lcn2 decreases the effect of surface proteins on TRAP activity and F-actin ring formation. Inhibition of lcn2 expression was measured by qPCR (**A**). RANKL-induced osteoclast formation was measured by TRAP staining (100 × , scale bar = 100 μm); (**B**) the number of osteoclasts were counted (**C**) and TRAP activity was quantified (**D**). RANKL-induced F-actin formation (green, white arrow) was measured by phalloidin staining (600 ×, scale bar = 40 μm) (**E**).

To demonstrate the interaction between lcn2 expression and TRAP activity in cells treated with surface proteins, lcn2 expression was inhibited via lcn2-siRNA transfection and TRAP activity was measured. The mRNA expression level of lcn2 significantly decreased in the lcn2-siRNA transfected cells compared with that in the control-siRNA-transfected cells (Fig. 4A). Following RANKL induction, TRAP activity and TRAP(+) osteoclast number significantly increased in the lcn2-siRNA transfected cells treated with surface proteins compared with that in the control siRNA-transfected cells treated with surface proteins (Fig. 4B–D). Furthermore, F-actin ring formation, which is responsible for bone resorption in mature osteoclasts, was measured by phalloidin staining. Upon RANKL treatment, an actin ring was formed along the inner side of large osteoclasts. The formation of actin-rich sealing zones was elevated in the lcn2-siRNA transfected cells treated with surface proteins compared with the control siRNA-transfected cells treated with surface proteins (Fig. 4E). These results suggest that surface proteins inhibit osteoclast differentiation by upregulating lcn2 expression.

### Inhibition of lcn2 decreases the inhibitory effects of surface proteins on the expression levels of osteoclast-related genes and proteins

**Figure 5:**
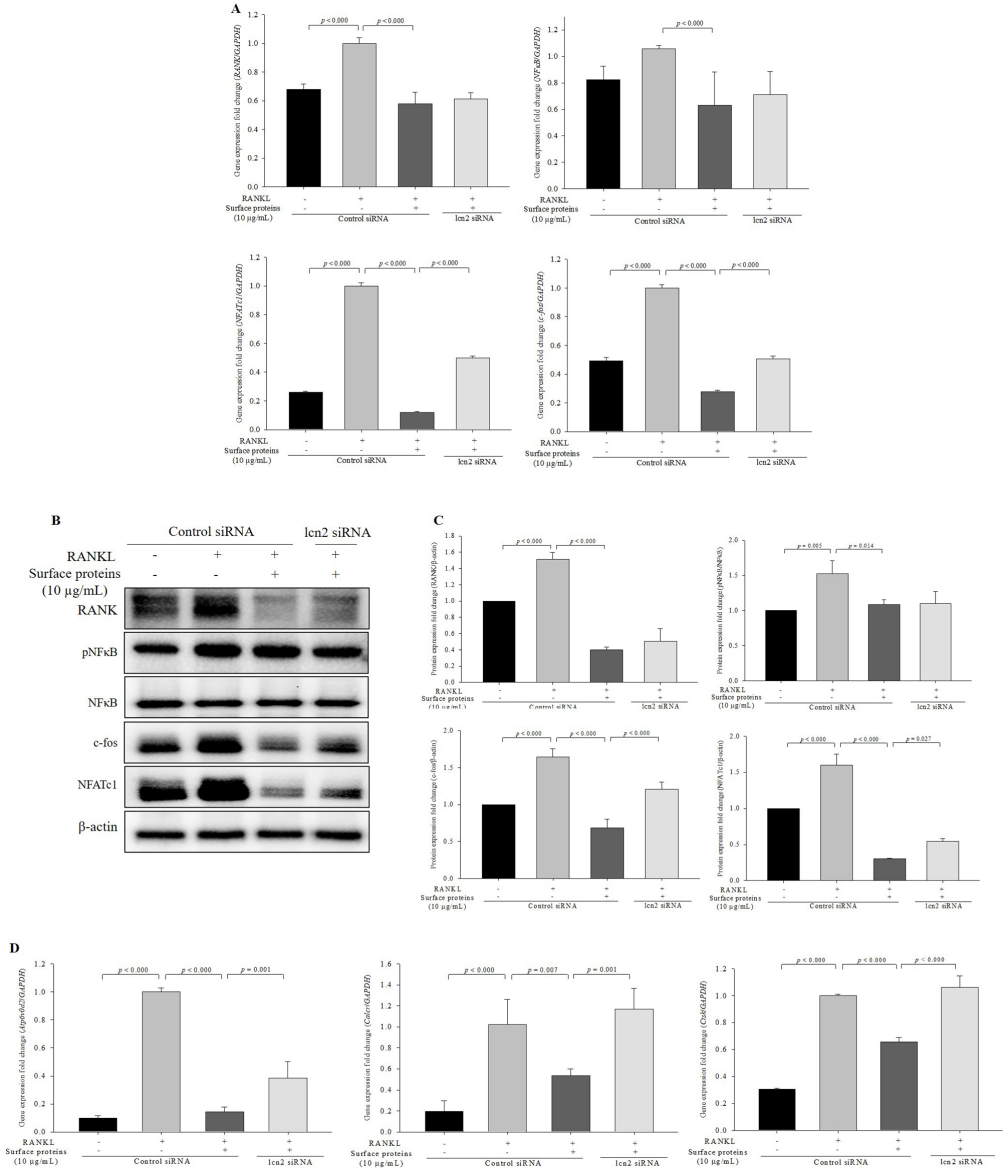
Inhibition of lcn2 decreases the effect of surface proteins on the expression levels of osteoclast-related genes and proteins. The expression levels of RANKL-induced osteoclastogenic genes measured by qPCR (**A**), expression levels of RANKL-induced osteoclastogenic proteins measured by western blotting (**B**) and quantified (**C**), and expression levels of NFATc1-downstream genes measured by qPCR (**D**). The values indicate the mean ± SD of three independent experiments performed in triplicates. The full-length blots are shown in Supplementary Fig. 5.

The expression levels of *c-fos* and *NFATc1* significantly increased in the lcn2-siRNA transfected cells treated with surface proteins compared with that in the control-siRNA transfected cells treated with surface proteins; however, the expression levels of *RANK* and *NFκB* genes were not increased in the lcn2-siRNA transfected cells (Fig. 5A). Likewise, the expression levels of c-fos and NFATc1 significantly increased in the lcn2-siRNA transfected cells treated with surface proteins compared with the those in the control siRNA-transfected cells treated with surface proteins at the protein level (Fig. 5B,C); with the expression levels of RANK and NFκB unaffected by lcn2 inhibition. Expression levels of NFATc1-downstream genes, *Atp6v0d2*, *Calcr,* and *Ctsk*, were significantly increased in the lcn2-siRNA transfected cells treated with surface proteins compared with those in the control-siRNA transfected cells treated with surface proteins (Fig. 5D). These results suggest that surface proteins inhibit osteoclast differentiation by upregulating lcn2 expression, which downregulates c-fos and NFATc1, followed by inhibition of the expression of NFATc1-downstream genes, *Atp6v0d2*, *Calcr,* and *Ctsk*.

### Heat-treated surface proteins and trypsin-shaved peptides from the bacterial cell surfaces inhibit osteoclastogenesis

**Figure 6:**
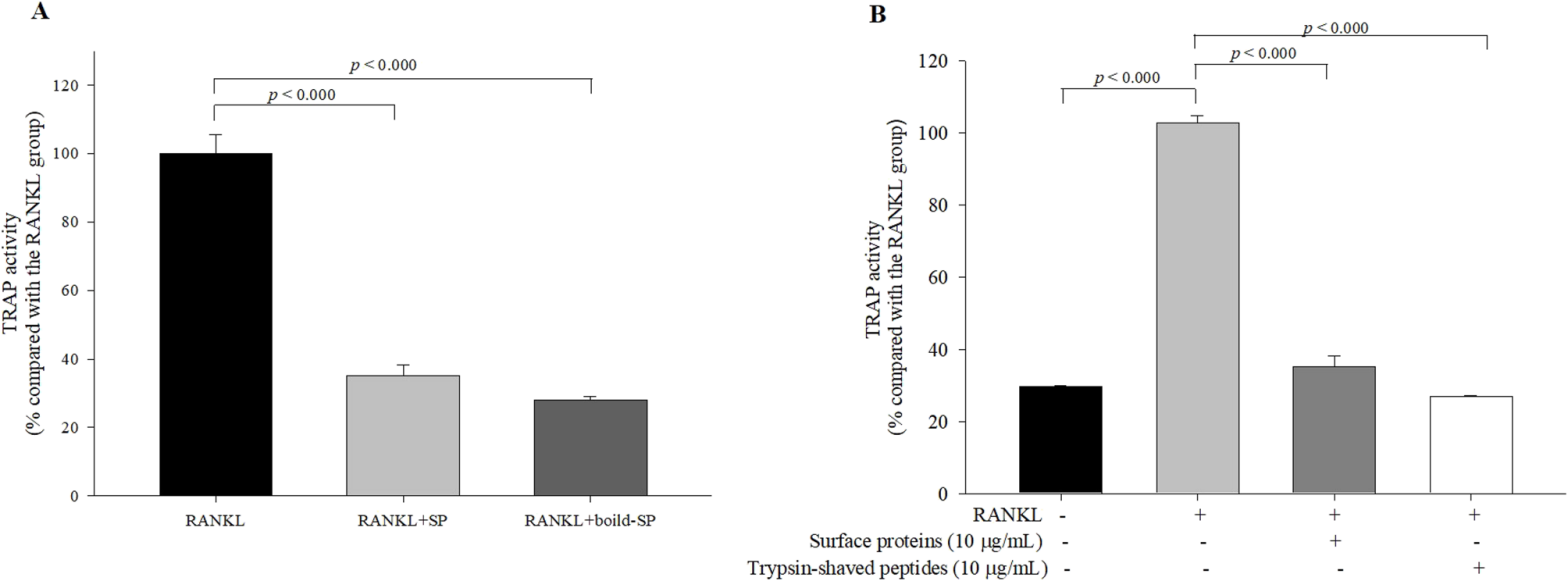
Effects of heat-treated-surface proteins and trypsin-shaved peptides on osteoclast differentiation. RANKL-induced osteoclast formation was measured using TRAP activity assay. TRAP activities of RANKL-induced cells treated with heat-treated surface proteins (10 μg/mL) (**A**) and trypsin-shaved peptides (**B**) were measured. The values indicate the mean ± SD of three independent experiments performed in triplicates.

In our previous study, heat-killed MJ2 at 100 °C for 30 min showed inhibitory effect on osteoclast differentiation^16^. Thus, the effect of heat-treated surface proteins on osteoclastogenesis was observed by measuring TRAP activity to investigate whether it showed an effect similar to that of non-heat-treated surface proteins. Similar to the effect of non-heat-treated surface proteins, TRAP activity in the cells treated with heat-treated surface proteins significantly decreased compared with that in the RANKL-only treated group (Fig. 6A). In addition, we investigated whether trypsin-shaved peptides from bacterial cell surfaces could inhibit osteoclastogenesis. Hence, we prepared trypsin-shaved peptides from the bacterial cell surfaces. TRAP activity in trypsin-shaved peptide-treated cells was also significantly decreased compared with that in the RANKL-only treated group (Fig. 6B). The inhibitory effect of trypsin-shaved peptides was similar to or slightly greater than that of surface proteins. Therefore, the inhibitory effect of surface proteins on osteoclastogenesis may not be derived from whole surface proteins.

### Identification of surface proteins extracted from *P. freudenreichii* MJ2

To identify the surface proteins from MJ2, Gu-HCl-extracted proteins were digested with trypsin and LC–MS/MS was performed. A list of proteins that matched more than five unique peptides and a score > 50 from *P. freudenreichii*-derived proteins was obtained by searching the MS/MS spectra (Table 2). Interestingly, chaperonins and heat shock proteins were the main Gu-HCl-extracted surface proteins.

**Table 2:** Surface proteins identified by LC–MS/MS. *PSMs* peptide spectrum matches; *MW* molecular weight; *pI* isoelectric point; Coverage, the number of amino acids in a specific protein sequence that are found in the peptides sequenced in MS/MS study.

Accession no.	Description	Coverage [%]	No. PSMs	No. unique peptides	MW [kDa]	pI	Score
A0A160VNM1	60 kDa chaperonin	59	77	22	56.2	4.83	351.76
D7GFM9	60 kDa chaperonin	42	56	17	56.5	4.82	210.54
A0A068VPD3	10 kDa chaperonin	60	39	5	10.6	5.05	149.58
A0A0A8S4V0	Heat shock protein 20 2 (20 kDa chaperone 2)	64	26	7	16.8	5.14	115.24
D7GI84	CsbD domain-containing protein	68	26	6	7.4	5.15	90.73
A0A068VTT9	Cold shock-like protein CspA	85	21	5	7.4	5	77.74
A0A2C8B4H7	Glyceraldehyde-3-phosphate dehydrogenase	26	26	10	37.8	5.73	72.68
A0A160VNJ1	Malate dehydrogenase	20	19	6	34.9	5.1	67.93

## Discussion

The surface layer components of some probiotic gram-positive bacteria, mainly proteins, glycoproteins, and lipoteichoic acids, have been studied for their probiotic effects, such as anti-inflammatory activity and inhibition of pathogen infection^19^. Surface proteins from *P. freudenreichii* have immunomodulatory properties in peripheral blood mononuclear cells and involve in adhesion to colon cells^14,20^. Surface proteins show immunomodulatory effects in the intestine^21,22^, which suggests that surface proteins obtained from probiotics may be developed as a treatment for immune-related diseases, such as inflammation, after evaluating their clinical effects. In this study, surface proteins of MJ2 inhibited the expression of *NFATc1* and also of *Ctsk*, *Calcr*, and *Atp6vod2* which engage in bone resorption by osteoclast^23^. TRAP and F-actin staining showed that surface proteins decreased the formation of mature osteoclasts. Thus, surface proteins of MJ2 showed inhibitory effect on osteoclast differentiation, suggesting that surface proteins of MJ2 might be developed as a treatment of RA.

Lcn2 is a member of the lipocalin family that transport small molecules^24^. Lcn2 acts as a bacteriostatic agent by sequestering siderophores, which are required for iron acquisition, and lcn2 depletion leads to increased susceptibility to bacterial infection^25^. Lcn2 plays a role in inflammation, cell differentiation, and lipid metabolism and is linked closely to osteoblast and osteoclast differentiation^18^. In bone marrow-derived macrophages, lcn2 overexpression results in a reduction in osteoclast differentiation. In contrast, lcn2-deleted mice showed a lower osteoblast number and subsequently decreased bone formation, and the conditioned medium of osteoblasts transfected with the lcn2-expression-vector showed an increase in osteoclastogenesis by inducing the pro-osteoclastogenic factor, RANKL^24^. Interestingly, lcn2 itself does not directly induce osteoclast formation; further, lcn2 suppresses osteoclast formation and differentiation by inhibiting the expression of c-fos and NFATc1 activation in isolated bone marrow cells^18,24,26^. In this study, treatment with surface proteins significantly increased the expression level of *lcn2*, and transfection with lcn2-siRNA diminished the inhibitory effect of surface proteins on the expression of genes and proteins related to osteoclastogenesis. Thus, surface proteins from MJ2 inhibit osteoclast differentiation by upregulating lcn2 expression. Lcn2 inhibits the NF-κB signaling pathway and RANKL-induced osteoclast differentiation^18^. In this study, however, although the expression level of *NFκB* and activation of NF-κB decreased in surface protein-treated cells, this change was not related to overexpression of lcn2, which is a negative regulator of NF-κB. In addition, the expression level of RANK was not affected by the overexpression of lcn2 in surface protein-treated cells; nevertheless, interestingly, NFATc1 and c-fos were affected by the overexpression of lcn2 in surface protein-treated cells. Further, in the microarray analysis, the expression level of *c-fos* was not significantly decreased; thus, we could not identify a direct association with lcn2 in this study. Consequently, we concluded that surface proteins from MJ2 may inhibit osteoclast differentiation signaling by inhibiting lcn2-mediated NFATc1 expression, but not by the NF-κB pathway.

The bacterial cell wall-extracted fraction or surface proteins include proteins that are believed to exist in the cytoplasm^27,28^. Likewise, Gu-HCl-extracted surface proteins from MJ2 include chaperonins and heat shock proteins, which are generally located in the bacterial cytoplasm. We could confirm that cell lysis did not occur during guanidine extraction; hence, we concluded that they might belong to moonlighting proteins. Moonlighting proteins are canonically known as cytoplasmic proteins that perform more than one distinct biochemical or biophysical functions that are not because of gene fusion or multiple proteolytic fragments^29^. In particular, heat-shock proteins (also known as chaperonins), typical surface moonlighting proteins, are present among the surface proteins of MJ2. Although extracellular heat-shock proteins (HSPs) play a role in immune response^27^, the functions of moonlighting proteins are still unknown, and approximately hundred moonlighting proteins have been identified and listed in the online MoonProt Database (moonlightingproteins.org). These proteins are type of surface proteins that do not have motif for surface export or anchoring and are referred to as anchorless proteins^30,31^. Moonlighting proteins mainly act as enzymes inside the cell, but they are also known for their adhesion function on the surface, including binding to plasminogen, extracellular matrix, or host cell surface proteins^32^. Because of these characteristics, moonlighting proteins of pathogens induce infection; in contrast, moonlighting proteins of probiotics bind to host mucin and make them colonize the gut^33^. In this study, we found that surface proteins of MJ2 and trypsin-cleaved peptides showed similar inhibitory effects on osteoclast differentiation, suggesting that the inhibitory activity of surface proteins on osteoclast differentiation may not be derived from whole surface proteins but from trypsin-cleaved peptides, functioning as moonlighting peptides.

In conclusion, surface proteins extracted from MJ2 showed inhibitory effects on osteoclast differentiation by upregulating lcn2. Overexpression of lcn2 by treatment with the surface proteins of MJ2 inhibits the expression of NFATc1, which in turn inhibits the expression of its downstream genes related to osteoclast differentiation. Thus, surface proteins inhibit osteoclastogenesis by mainly upregulating lcn2, which subsequently downregulates NFATc1, leading to the inhibition of expression of NFATc1-downstream genes related to osteoclast differentiation. Although we found that the surface proteins of MJ2 inhibited osteoclast formation from murine macrophage RAW 264.7, we need to investigate the inhibitory effects of the surface proteins on osteoclast differentiation from bone marrow-derived macrophages (BMM) in vitro and inflammatory-induced osteolysis in vivo. Surface proteins from other bacteria may or may not show inhibitory effect on osteoclastogenesis, therefore, extensive study on inhibitory effect on osteoclastogenesis of surface proteins of bacterial strains is necessary. Further, the identification of the main protein(s) in the surface proteins is necessary to elucidate the mechanism by which surface proteins from MJ2 inhibit osteoclast differentiation and the identification of the receptor(s) of the main protein(s) in BMM or osteoclast involved in the inhibition of osteoclast formation is needed in our further studies.

## Materials and methods

### Materials

Minimum Essential Medium-α Modification (α-MEM), Dulbecco’s Modified Eagle Medium (DMEM), fetal bovine serum (FBS), and penicillin/streptomycin for the cultivation of cells were purchased from Hyclone (Logan, UT, USA). MTT (3-[4,5-dimethylthiazol-2-yl]-2,5-diphenyltetrazolium bromide) was obtained from Amresco (Solon, OH, USA). Recombinant murine RANK Ligand (RANKL) was purchased from Peprotech (Rocky Hill, NJ, USA). Gu-HCl was purchased from Sigma-Aldrich (St. Louis, MO, USA). Lipofectamine RNAiMAX Transfection Reagent was purchased from Thermo Fisher Scientific (Waltham, MA, USA).

### Preparation of surface proteins from *P. freudenreichii* MJ2

*P. freudenreichii* MJ2 (KCCM12272P)^15^ was cultured in reinforced clostridial medium (RCM) at 30 °C for 48 h under anaerobic conditions. After incubation, *P. freudenreichii* MJ2 cells were harvested by centrifugation at 4000 × *g* for 5 min at 4 °C and then washed twice with 20 mM Tris–HCl buffer to remove the salts and soft agar in the medium. Bacterial cells were washed with phosphate-buffered saline (PBS), and the pelleted cells were incubated in 5 M Gu-HCl for 30 min and centrifuged at 10,000 × *g* for 10 min at 4 °C. To isolate the surface proteins, the supernatant was separated and dialyzed using a dialysis tubing cellulose membrane (Sigma-Aldrich). The surface proteins were quantified using the Bradford method, and bovine serum albumin (BSA) was used as the standard. Collected surface proteins were diluted in PBS and stored at − 20 °C.

### Cell culture and cytotoxicity assay

The murine macrophage cell line RAW 264.7 from the Korea Cell Line Bank (KCLB, Seoul, Korea) were grown in DMEM with 10% FBS, penicillin (100 U/mL), and streptomycin (100 μg/mL) at 37 °C with 5% CO_2_. RAW 264.7 cells (1 × 10^4^ cells/mL) were seeded in 96-well plates. After 24 h, the cells were treated with the extracted surface proteins (2.5, 5, or 10 μg/mL) for 4 days. By MTT assay, cell viability was measured at 540 nm using a SpectraMax 340PC384 plate reader (Molecular Devices, Sunnyvale, CA, USA) and was calculated as the percentage relative to the measure of negative control.

### Carboxyfluorescein diacetate (CFDA)/propidium iodide (PI) staining

A 24-well plate was coated with sterilized coverslips with 0.1% gelatin, and MJ2 suspension was diluted to 1 × 10^6^ CFU/mL in PBS and inoculated into the plate. After overnight incubation, bacterial cells were washed with PBS and stained with 100 μM CFDA solution in the dark for 15 min at 37 °C. The cells were then incubated with a 15 μM PI solution for 10 min. Bacterial cells were washed with PBS and fixed with 4% formaldehyde. Staining analysis was performed using a Nikon C1 plus confocal scanning microscope with laser light at 488 and 620 nm.

### Tartrate-resistant acid phosphatase (TRAP) staining and activity assay

RAW 264.7 cells (1 × 10^4^ cells/mL) were seeded in 24-well plates and were incubated at 37 °C overnight. For osteoclast differentiation, the medium was replaced with α-MEM and the cells were treated with RANKL (50 ng/mL) and the extracted surface proteins (2.5, 5, or 10 μg/mL) for 4 days. The cells were stained using a TRAP staining kit (Takara Biotechnology, Shiga, Japan), according to the manufacturer’s protocol. TRAP-positive multinucleated cells containing more than three nuclei were counted under a light microscope. TRAP activity was assayed as previously described^34^. Briefly, the fixed cells were incubated with 50 mM citrate buffer (pH 4.5) containing 10 mM sodium tartrate and 6 mM *p*-nitrophenylphosphate for 1 h at 37 °C and the reaction was stopped by adding an equal volume of 0.1 N NaOH solution, The optical density was measured at 405 nm. Activity was expressed as the percentage of the value obtained for negative control.

### Quantitative real-time polymerase chain reaction (qPCR)

Total RNA was extracted using Ribo-Ex reagent (GeneAll Biotechnology, Seoul, Korea). qPCR was performed with cDNA converted with a RevertAid First Strand cDNA Synthesis kit (Thermo Fisher Scientific) using a Kapa SYBR Fast qPCR kit (Kapa Biosystems, Woburn, MA, USA) and a 7500 Fast Real-Time PCR system (Applied Biosystems, Foster City, CA, USA). The reaction was preheated to 95 °C for 10 min, followed by 40 cycles at 95 °C for 15 s, 60 °C for 15 s, and 72 °C for 30 s. *GAPDH* was used as the reference gene, and the primer sequences used in this study are shown in Supplementary Table 1. Relative gene expression was quantified based on equal amounts of RNA. The normalized expression change was expressed as 2^−ΔΔCt^ (with the value of *GAPDH* control set to 1)^35^.

### Western blotting

RAW 264.7 cells (1 × 10^4^ cells/mL) were seeded in 24-well and were incubated at 37 °C overnight and then the medium was replaced with α-MEM. The cells were treated with RANKL (50 ng/mL) and the extracted surface proteins (2.5, 5, or 10 μg/mL). The cells were harvested, and total protein was extracted using radioimmunoprecipitation assay (RIPA) buffer (Rockland Immunochemicals, Limerick, PA, USA) containing Halt™ protease inhibitor cocktail (Thermo Fisher Scientific). The total protein content was quantified by the Bradford method. Equal amounts of protein (10‒20 μg) were denatured and separated by 10% sodium dodecyl sulfate–polyacrylamide gel electrophoresis (SDS-PAGE). Proteins were transferred to PVDF membranes (Millipore, Bedford, MA, USA) and blocked with 5% dry nonfat skim milk in Tris-buffered saline with 0.05% Tween 20 (TBST) for 2 h. After washing with TBST, the membranes were incubated with primary antibodies at 4 °C overnight. Antibodies against NFATc1 (1:1000 dilution, BD556602; BD Biosciences), c-fos (1:1000 dilution, 2250S; Cell Signaling), RANK (1:500 dilution, sc-374360; Santa Cruz), phosphorylated p65 NF-κB (1:1000 dilution, S536; Cell Signaling), p65 NF-κB (1:1000 dilution, 8242S; Cell Signaling), and the endogenous control β-actin (1:5000 dilution, MA5-15739; Thermo Fisher Scientific) were used. After washing with TBST three times, the membrane was incubated with secondary antibody for 1 h at room temperature. Goat anti-mouse IgG (H + L) horseradish peroxidase (HRP)-conjugated antibody (1:10,000 dilution, NCI1430KR; Thermo Fisher Scientific) was used for anti-NFATc1, RANK, and β-actin detection, and goat anti-rabbit IgG (H + L) horseradish peroxidase-conjugated antibody (1:5000 dilution, NCI1460KR; Thermo Fisher Scientific) was used to detect all the other proteins. After washing with TBST, the membrane was developed using the SuperSignal West Femto Maximum Sensitivity Substrate kit (Thermo Fisher Scientific). Images were captured with a FluorChem E System (Protein Simple, California, USA) and quantified using ImageJ Gel Analysis program (Softomic, Barcelona, Spain).

### Microarray analysis

Total RNA from the negative control and surface protein-treated cells was extracted with RiboEx (GeneAll Biotechnology) according to the manufacturer’s instructions. RNA purity and integrity were measured using a NanoDrop ND-1000 spectrophotometer (Thermo Fisher Scientific) and an Agilent 2100 Bioanalyzer (Agilent Technologies, Santa Clara, CA, USA), respectively. cDNA libraries were constructed using a QuantSeq 3′ mRNA-Seq Library Prep Kit (Lexogen, Greenland, NH, USA) according to the manufacturer’s instructions, and 75 bp single-end sequencing was performed on an Illumina NextSeq 500 platform (Illumina, San Diego, CA, USA). All data were obtained after quantile normalization between samples, and differentially expressed genes (DEGs) were identified using Excel-based Differentially Expressed Gene Analysis (ExDEGA) version 2.0.0 provided by eBiogen (Seoul, Korea). Genes with a *p* value < 0.05 and a fold change of 2.0, compared with the gene expression in the negative control sample, were identified as significantly differentially expressed genes. RNA sequencing data have been deposited in the NCBI Gene Expression Omnibus (GEO) database (accession number: GSE213113). The verified genes were analyzed using the KEGG (database and visualized using a heatmap). The relationship between each gene was visualized using the STRING analysis tool under the minimum required interaction score setting, with a medium confidence of 0.400.

### Small interfering RNA (siRNA) transfection

RAW 264.7 cells were seeded in 12-well plates at a density of 1 × 10^5^ cells/mL without antibiotics. After incubation at 37 °C overnight, the medium was changed to α-MEM without serum or antibiotics. For each siRNA transfection (Bioneer), non-correlated siRNA or lipocalin-2 (lcn2)-siRNA (final concentration of 100 nM) was combined with an equal volume of Lipofectamine RNAiMAX reagent (Thermo Fisher Scientific). The sequences of the lcn2-siRNA duplex were duplex 1 (5′-GCA CAG GUA UCC UCA GGU A-3′) and duplex 2 (5′-UAC CUG AGG AUA CCU GUG C-3′). The mixture was incubated for 20 min at room temperature and added to each well. The cells were incubated for 6 h and treated with RANKL (50 ng/mL) and the extracted surface proteins (2.5, 5, or 10 μg/mL) in the absence of antibiotics. Transfection efficiency was verified by confirming that the lcn2 gene expression level was decreased after siRNA transfection (Supplementary Fig. 3).

### F-actin staining

RAW 264.7 cells were seeded on coverslips with 0.1% gelatin in 24-well plates at a density of 1 × 10^4^ cells/mL. After incubation at 37 °C overnight, the medium was changed to α-MEM. The cells were transfected with lcn2-siRNA or control siRNA and treated with RANKL (50 ng/mL) and the extracted surface proteins (10 μg/mL) for 4 days. The cells were washed, fixed, and stained using phalloidin-iFluor 488 reagent (Abcam, Cambridge, UK). Nuclei were also stained with 4′,6′-diamidino-2-phenylindole (DAPI), and the coverslips were mounted on glass slides. Images were captured using a C1 Plus confocal laser scanning microscope (Nikon, Tokyo, Japan).

### Enzymatic shaving of surface proteins

*P. freudenreichii* MJ2 was cultured in RCM broth and washed in PBS containing 5 mM dithiothreitol (DTT) after centrifugation. The bacteria were suspended in sequencing-grade modified trypsin (V5111; Promega, Madison, WI, USA). Specifically, the enzymatic shaving was performed by incubating 5 × 10^9^ CFU/mL of bacteria and 4 μg of trypsin together for 1 h at 37 °C with gentle shaking conditions. Bacterial cells were removed by centrifugation at 10,000 × *g* for 10 min at 4 °C, and the supernatant was collected for trypsin-shaved samples.

### Protein identification by LC–MS/MS

Surface proteins (0.5 mg/mL) were reduced by 5 mM DTT at 56 °C for 30 min and alkylated with iodoacetamide (IAA) at room temperature for 45 min. Proteins were digested with 4 μg of sequencing-grade modified porcine trypsin (Promega) and incubated overnight at 37 °C. The peptides were separated using a Vanquish HPLC system (Thermo Fisher Scientific) and a Hypersil GOLD C18 (2.1 × 50 mm, 1.9 μm, Thermo Fisher Scientific) column. Mobile phase A was water with 0.2% (v/v) formic acid and mobile phase B was 0.2% (v/v) formic acid in acetonitrile. The peptides were separated using a gradient step. The eluted peptides were analyzed using Q Exactive Plus (Thermo Fisher Scientific). The mass resolution was set to 70,000, and measurements were taken in full scan/ddMS2 mode (full scan data dependent MS/MS) in the range of 200–2000 m/z. Peptide identification from raw data was carried out using Proteome Discoverer program (ver 2.4), and the following parameters were used: tryptic cleavage up to 2 missed cleavage sites, tolerances of 10 ppm for precursor mass tolerance, and 0.02 Da for fragment mass tolerance. The identified peptides were tested for specificity and sequence homology using the NCBI database.

### Statistical analysis

Statistical analyses were performed using SPSS 24.0 (SPSS Inc., Chicago, IL, USA). Differences between two groups were tested using Student’s *t* test, and differences among multiple groups were determined by one-way analysis of variance (ANOVA) followed by Tukey’s honestly significant difference (HSD) post-hoc test. The experimental values were expressed as mean ± standard deviation (SD). A *p* value of < 0.05 was considered statistically significant.

### Supplementary Information

Supplementary Information.

## Data Availability

All generated data have been included in the published manuscript. The datasets of gene expression generated during the current study are available in the NCBI Gene Expression Omnibus (GEO) database (accession number: GSE213113). For all other information, contact with the corresponding author (Young-Hee Lim. yhlim@korea.ac.kr).
