# Supplementary material for: Surface proteins of Propionibacterium freudenreichii MJ2 inhibit RANKL-induced osteoclast differentiation by lipocalin-2 upregulation and lipocalin-2-mediated NFATc1 inhibition
Source: Sci Rep. 2023 Sep 20;13:15644. doi: 10.1038/s41598-023-42944-y (PMC10511438; doi:10.1038/s41598-023-42944-y)
Supplement: Supplementary file 1 — Supplementary Information. [file 41598_2023_42944_MOESM1_ESM.pdf]

Supplementary Information

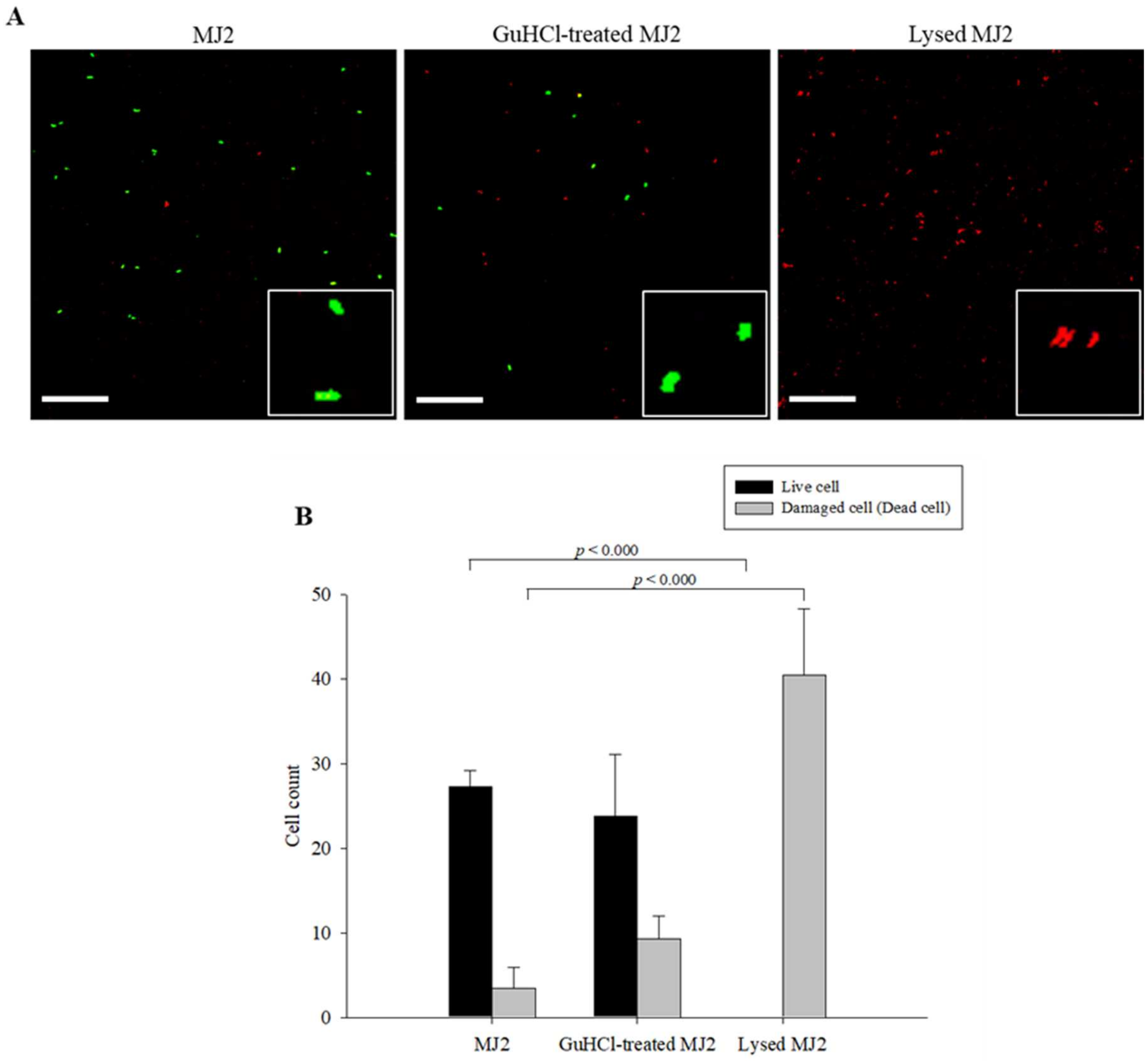

Supplementary Fig. 1. Viability staining on *P. freudenreichii* MJ2 after extracting with guanidine-HCl (Gu-HCl) using carboxyfluorescein diacetate (CFDA)/propidium iodide (PI). Gu-HCl extracted *P. freudenreichii* MJ2 stained with CFDA/PI solution (A) and quantified (B) (1000×, scale bar = 10 μm). Lysed MJ2 was prepared using RIPA buffer (Rockland Immunochemicals, Limerick, PA, USA) containing Halt™ protease inhibitor cocktail (Thermo Fisher Scientific). The values indicate the mean ± SD of three independent experiments performed in triplicates.

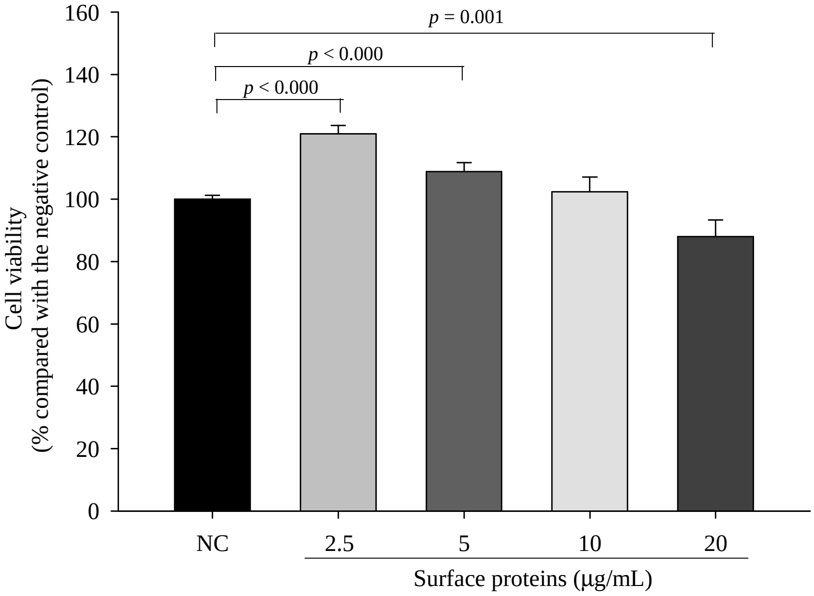

Supplementary Fig. 2. Cytotoxicity of surface proteins extracted from *P. freudenreichii* MJ2 on raw 264.7 cells. The viability was measured by MTT assay. The values indicate the mean ± SD of three independent experiments performed in triplicates.

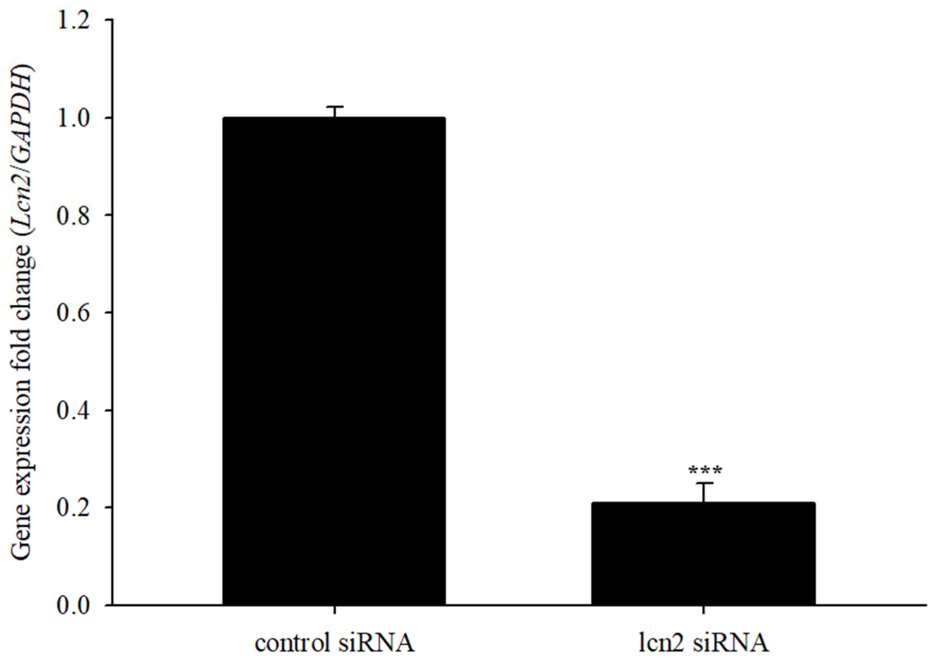

Supplementary Fig. 3. Knockdown efficacy of lcn2 siRNAs. Raw 264.7 cells were transfected with 100 nM siRNAs and after 24 h the total RNA was isolated and quantitative real-time PCR was performed. Data are shown as the mean ± SD of three independent experiments performed in triplicate. Student's *t*-test was used to determine the significance of the differences. ***$p < 0.001$.

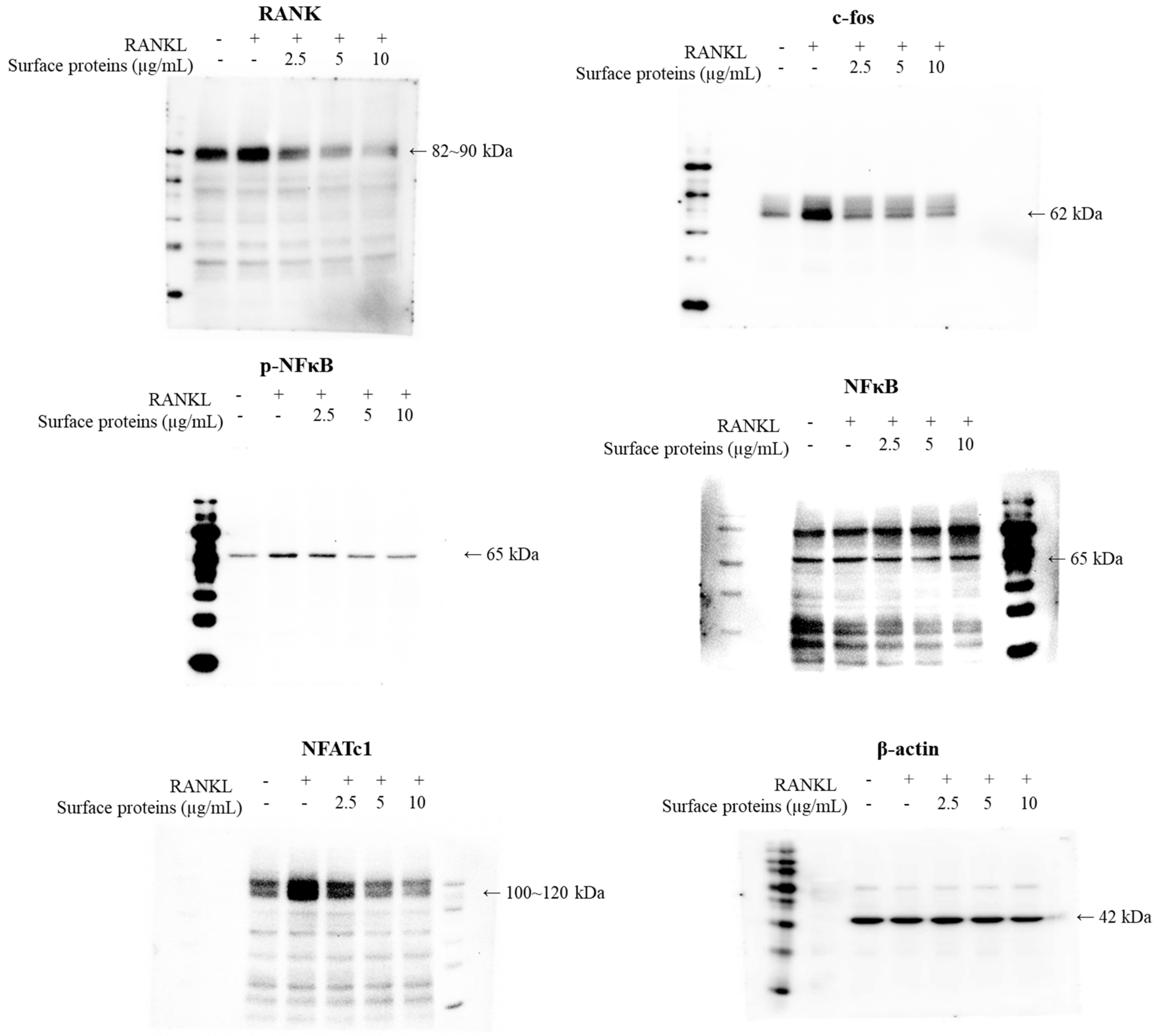

Supplementary Fig. 4. The full-length blots of the proteins shown in in Fig. 2B.

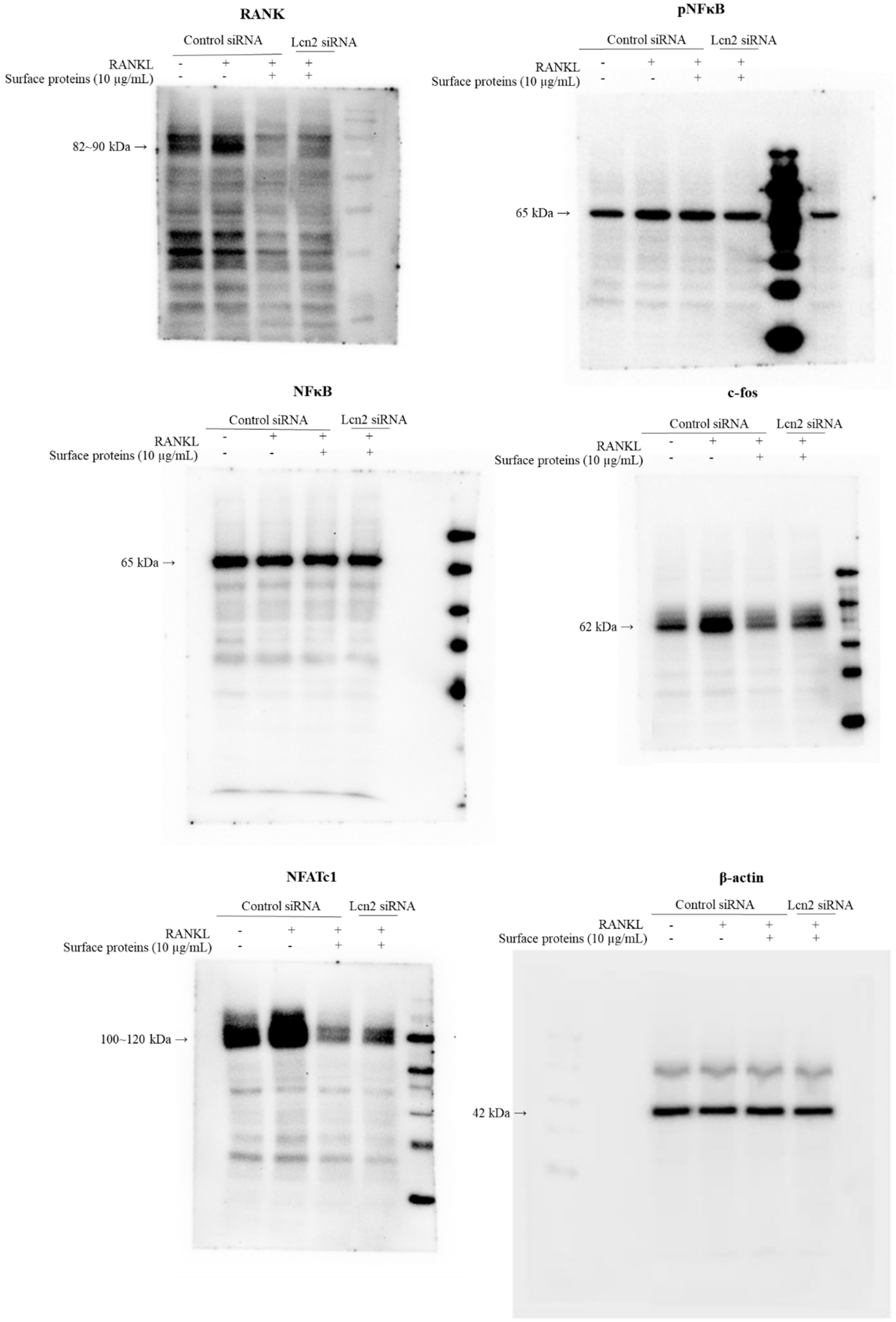

Supplementary Fig. 5. The full-length blots of the proteins shown in in Fig. 5B.

Supplementary Table 1. Primer sequences used for qPCR.

Gene	Forward (5′-3′)	Reverse (5′-3′)
GAPDH	ACCCAGAAGACTGTGGATGG	CACATTGGGGGTAGGAACAC
RANK	TGCAGCTCAACAAGGATACG	GAGCTGCAGACCACATCTGA
NFκB	TCCTGGCCTCTAGCCTTGTA	GCCAAGGAAGAAAAGTGCTG
c-fos	CCAGTCAAGAGCATCAGCAA	AAGTAGTGCAGCCCGGAGTA
NFATc1	GGTGCTGTCTGGCCATAACT	GCGGAAAGGTGGTATCTCAA
Atp6v0d2	GACCCTGTGGCACTTTTTGT	GCTTGCATTTGGGGAATCTA
Calcr	CGGACTTTGACACAGCAGAA	GTCACCCTCTGGCAGCTAAG
Ctsk	CAGCTTCCCCAAGATGTGAT	AGCACCAACGAGAGGAGAAA
Lcn2	TGGAAGAACCAAGGAGCTGT	GATGGAGTGGCAGACAGACA
